# Author Correction: Constraining the rise of oxygen with oxygen isotopes

**DOI:** 10.1038/s41467-020-18888-6

**Published:** 2020-09-30

**Authors:** B. A. Killingsworth, P. Sansjofre, P. Philippot, P. Cartigny, C. Thomazo, S. V. Lalonde

**Affiliations:** 1 CNRS-UMR6538 Laboratoire Géosciences Océan, European Institute for Marine Studies, Université de Bretagne Occidentale, 29280 Plouzané, France; 2 Géosciences Montpellier, CNRS-UMR 5243, Université de Montpellier, Montpellier Cedex 5, France; 3 Institut de Physique du Globe de Paris, Sorbonne-Paris Cité, UMR 7154, CNRS-Université Paris Diderot, 75005 Paris Cedex 05, France; 4 UMR CNRS/uB 6282 Laboratoire Biogéosciences, Université de Bourgogne Franche-Comté, 6 Bd Gabriel, 21000 Dijon, France; 5 Present Address: Institut de Physique du Globe de Paris, Sorbonne-Paris Cité, UMR 7154, CNRS-Université Paris Diderot, 75005 Paris Cedex 05, France; 6 Present Address: Muséum d’Histoire Naturelle, Sorbonne Université, UMR CNRS 7590, Institut de Minéralogie, de Physique des Matériaux et de Cosmochimie, 75005 Paris, France

**Keywords:** Element cycles, Atmospheric chemistry, Palaeoceanography, Marine chemistry

Correction to: *Nature Communications* 10.1038/s41467-019-12883-2, published online 29 October 2019.

The original version of this Article contained an error in the Source Data for Fig. 1a. This caused some of the sulfate ∆33S data (in dark blue open diamond symbols) from around ~2.5 to 2.6 Ga (horizontal axis) to be displayed incorrectly in Fig. 1a. The caption of Fig. 1 remains the same and the mistake does not affect the conclusions of the paper. The Source Data file and Fig. 1a have now been corrected in the PDF and HTML versions of the Article.

