# Supplementary material for: Constraining the rise of oxygen with oxygen isotopes
Source: Nat Commun. 2019 Oct 29;10:4924. doi: 10.1038/s41467-019-12883-2 (PMC6820740; doi:10.1038/s41467-019-12883-2)
Supplement: Supplementary file 2 — Supplementary Information [file 41467_2019_12883_MOESM2_ESM.docx]

Supplementary Information for

**Constraining the rise of oxygen with oxygen isotopes**

Killingsworth et al.

correspondence to: [bryan.a.killingsworth@gmail.com](mailto:bryan.a.killingsworth@gmail.com)

**Supplementary Figure 1.** Geologic maps adapted in Philippot et al., 2018^1^, and included here under the terms of its Creative Commons license ([http://creativecommons.org/](http://creativecommons.org/ licenses/by/4.0/)

[licenses/by/4.0/](http://creativecommons.org/ licenses/by/4.0/)), showing the (a) geographic context of the Turee Creek Group, and (b) location of the three drill cores of the Turee Creek Group Drilling Project (TCDP), with the study core referred to as TCDP3 in the text here labelled on the map as T3.

**Supplementary Figure 2.** Core photographs from TCDP3 labelled with their depths. Fields of view are 4.5 by 10 cm. Starred images approximately correspond to thin sections in Supplementary Fig. 5. 183m: Koolbye Formation quartzite in lower core. 182.4m: synsedimentary faulting around Koolbye/Kazput contact. 182.2m: approximate to Fe and S enrichments. 181m: synsedimentary faulting. 179.5m: laminated muddy carbonated siltstone. 170.7m: finely laminated dark muddy siltstone overlain by more carbonated layered siltstone. 158.5m: more planar, regular laminations. 149m: just below increase in carbonate content. 135.2m carbonate with siliciclastic layering. 123.8m: carbonate near interval of peak δ^34^S compositions. 116.4m: near center of carbonate unit. 104.8m: carbonate. 97.8m: increasing siliciclastic layering, with breaks in core outlined on photo. 96m: carbonate with silicilastic layering. 84.3m: return to laminated carbonated siltstone. 77.1m: laminated carbonated siltstone near top of core.

**Supplementary Figure 3.** Photomicrograph and EDS spectra of barite from sample CAS-9 detected by scanning electron microscope (SEM). To concentrate the trace barites for imaging, decarbonated sample powder was digested with hydrofluoric acid and the residual material was dried and analysed by SEM.

**Supplementary Figure 4.** SEM photomicrograph and EDS spectra of barite in sample CAS-9.

**Supplementary Figure 5.** XRF scans and photomicrographs, in reflected light (RL) and plane polarized light (PPL), of thin sections from the TCDP3 drill core. Scale bars are given for the XRF scans, with Ca, S, and Al contents color-coded. Photomicrograph fields of view are 1.2 mm by 0.9 mm, with the exception of the one pair of photomicrographs with a scale bar.

** Supplementary Figure 6.** Slide scan image of petrographic thin section from 104.8 m depth in TCDP core 3, Kazput Formation. The approximate field of view is 20 x 40 mm and is oriented with stratigraphic up towards to the top of the image. The fine-grained and finely-laminated texture that is present throughout TCDP drill core 3 is observed in this thin section that is from the central shaley carbonate discussed in the main text. The black rectangular shape in the center-left is a euhedral pyrite.

**Supplementary Figure 7.** The oxygen isotope compositions (δ^18^O) are shown for dissolved sulphate in tap water, sulphate derived from the deliberate oxidation of pyrite in distilled laboratory water, and the average for barium sulphates extracted from TCDP core 3. The sulphate in tap water was precipitated from municipal water in the laboratory in Plouzané, France. The sulphate derived from the oxidation of pyrite was generated by stirring crushed and powdered pyrite for >1 day in laboratory distilled water, using the same distilled water source that was used in the barite extractant solutions.

**Supplementary Table 1.** Kazput barite multiple sulphur (∆^33^S, ∆^36^S, and δ^34^S) and oxygen (δ^18^O) isotope data.

| **sample** | **depth (m)** | **∆^33^S (‰)** | **∆^36^S (‰)** | **δ^34^S (‰ VCDT)** | **δ^18^O (‰ VSMOW)** |
| --- | --- | --- | --- | --- | --- |
| CAS-1 | 84.98 | 0.82 | -0.9 | 2.0 | - |
| CAS-3 | 87.60 | 0.70 | -1.0 | 2.9 | -15.0 |
| CAS-4 | 88.92 | 0.62 | -0.7 | 1.0 | -2.7 |
| CAS-5 | 90.53 | 0.76 | -1.2 | 4.8 | -9.4 |
| CAS-6 | 92.05 | 0.90 | -1.1 | 3.5 | -12.7 |
| CAS-8 | 95.04 | - | - | - | -12.7 |
| CAS-9 | 96.53 | - | - | - | -16.8 |
| CAS-10 | 97.50 | 1.11 | -1.6 | 5.0 | -13.8 |
| CAS-11 | 99.02 | - | - | - | -16.1 |
| CAS-13 | 102.15 | 1.42 | -1.9 | 5.4 | -18.0 |
| CAS-14 | 103.77 | 1.47 | -2.0 | 6.2 | -11.8 |
| CAS-15 | 104.97 | 1.48 | -1.7 | 6.4 | -15.2 |
| CAS-16 | 106.63 | - | - | - | -9.2 |
| CAS-18 | 109.64 | 1.37 | -1.6 | 7.6 | -8.9 |
| CAS-19 | 110.96 | 1.47 | -1.7 | 6.9 | -19.5 |
| CAS-20 | 112.58 | 1.37 | -1.4 | 6.9 | -3.9 |
| CAS-21 | 114.03 | 1.39 | -1.6 | 7.6 | -12.0 |
| CAS-23 | 116.99 | 1.29 | -1.7 | 5.6 | 2.2 |
| CAS-24 | 118.63 | 1.48 | -1.6 | 10.0 | -14.9 |
| CAS-26 | 121.58 | 1.43 | -1.7 | 10.7 | -10.8 |
| CAS-28 | 124.67 | 1.42 | -1.4 | 12.5 | -19.1 |
| CAS-29 | 126.09 | - | - | - | -13.5 |
| CAS-30 | 127.60 | 1.55 | -1.6 | 11.8 | -15.9 |
| CAS-31 | 128.92 | - | - | - | -11.0 |
| CAS-33 | 132.14 | 1.51 | -1.7 | 10.0 | -10.6 |
| CAS-34 | 133.61 | 1.37 | -1.5 | 8.3 | -10.3 |
| CAS-35 | 135.09 | 1.29 | -1.8 | 6.9 | -2.9 |
| CAS-36 | 136.51 | - | - | - | -7.0 |
| CAS-38 | 139.56 | 1.16 | -1.4 | 6.7 | -8.8 |
| CAS-39 | 141.23 | 0.98 | -1.3 | 5.8 | -13.7 |
| CAS-40 | 142.68 | 0.90 | -1.3 | 5.6 | -3.3 |
| CAS-41 | 144.12 | - | - | - | -2.0 |
| **MIN** |  | 0.62 | -2.0 | 1.0 | -19.5 |
| **MAX** |  | 1.55 | -0.7 | 12.5 | 2.2 |
| **AVERAGE** |  | 1.22 | -1.5 | 6.7 | -11.0 |

**Supplementary Table 2.** The weight percent carbonate for TCDP core 3 shown here is based on measurements performed by ACTLABS using alkaline fusion total digest of sample powders. The reported data for CaO oxide weight percent is added to loss on ignition (assumed to entirely represent CO_2_) to give the percentage of carbonate shown, as carbonates were not specifically measured for this study. This accuracy of this determination of carbonate weight percent was confirmed gravimetrically on one sample (CAS-23) by weighing dry sample powder, decarbonating it in acid and drying, then weighing the remaining dry sample reside. Weight percent S was previously reported in Philippot et al., 2018^1^, with the weight percent pyrite also done for that study but first reported here. Pyrite weight percent determinations were made gravimetrically from chromium reducible sulfide yields from bulk sample powders.

| **Depth (m)** | **wt. % carb.** | **wt. % S ^1^** | **wt. % pyrite (FeS_2_)** | **Spy/S** |
| --- | --- | --- | --- | --- |
| 76.29 | 7.75 | 0.07 |  |  |
| 79.19 | 7.94 | 0.10 |  |  |
| 82.52 | 11.53 | 0.07 |  |  |
| 85.30 | 4.24 | 0.15 |  |  |
| 86.34 | 46.15 | 0.24 |  |  |
| 89.11 | 52.28 | 0.19 |  |  |
| 91.86 | 51.77 | 0.20 |  |  |
| 93.50 | 56.85 | 0.20 | 0.19 | 0.25 |
| 96.01 | 49.06 | 0.24 |  |  |
| 98.48 | 43.47 | 0.21 |  |  |
| 100.57 | 39.35 | 0.17 | 0.46 | 0.73 |
| 102.67 | 46.47 | 0.22 |  |  |
| 103.41 | 45.69 | 0.20 |  |  |
| 105.25 | 57.25 | 0.24 | 0.17 | 0.19 |
| 107.81 | 59.45 | 0.23 |  |  |
| 108.69 | 55.91 | 0.22 |  |  |
| 109.80 | 44.07 | 0.21 | 0.55 | 0.71 |
| 115.14 | 54.02 | 0.23 |  |  |
| 116.42 | 39.87 | 0.22 |  |  |

| 117.77 | 84.17 | 0.21 |  |  |
| --- | --- | --- | --- | --- |
| 118.27 | 62.01 | 0.21 | 0.30 | 0.39 |
| 118.83 | 54.23 | 0.23 |  |  |
| 120.79 | 59.06 | 0.23 |  |  |
| 123.86 | 54.79 | 0.23 |  |  |
| 124.33 | 60.74 | 0.24 |  |  |
| 126.29 | 67.45 | 0.28 | 0.33 | 0.32 |
| 129.36 | 63.21 | 0.23 |  |  |
| 129.47 | 63.59 | 0.27 | 0.20 | 0.20 |
| 131.92 | 57.83 | 0.21 |  |  |
| 134.51 | 62.70 | 0.22 |  |  |
| 135.23 | 54.68 | 0.23 | 0.46 | 0.54 |
| 138.12 | 52.94 | 0.23 |  |  |
| 140.27 | 57.32 | 0.24 |  |  |
| 141.72 | 55.62 | 0.24 |  |  |
| 144.41 | 49.04 | 0.23 | 0.25 | 0.29 |
| 144.85 | 43.11 | 0.30 |  |  |
| 146.13 | 14.48 | 0.15 |  |  |
| 146.94 | 16.77 | 0.24 |  |  |
| 149.21 | 8.65 | 0.09 |  |  |
| 149.26 | 5.60 | 0.02 |  |  |
| 151.57 | 9.32 | 0.11 |  |  |
| 153.33 | 19.67 | 0.04 |  |  |
| 155.02 | 24.01 | 0.06 |  |  |
| 155.13 | 12.99 | 0.05 |  |  |
| 156.60 | 19.67 | 0.04 |  |  |
| 157.31 | 20.32 | 0.04 |  |  |
| 159.21 | 13.65 | 0.09 |  |  |
| 161.23 | 15.35 | 0.09 |  |  |
| 162.29 | 16.09 | 0.13 |  |  |
| 162.90 | 13.48 | 0.03 |  |  |
| 164.21 | 18.56 | 2.85 |  |  |
| 164.40 | 11.90 | 2.07 |  |  |
| 164.58 | 21.38 | 2.67 |  |  |
| 165.11 | 6.02 | 0.14 |  |  |
| 170.61 | 4.86 | 0.26 |  |  |
| 171.68 | 4.59 | 0.15 |  |  |
| 171.76 | 4.77 | 0.10 |  |  |
| 172.02 | 4.64 | 0.16 |  |  |
| 172.21 | 4.70 | 0.24 |  |  |
| 173.58 | 4.62 | 0.12 |  |  |
| 173.70 | 5.06 | 0.15 |  |  |
| 174.34 | 9.85 | 0.31 |  |  |
| 176.13 | 6.32 | 0.16 |  |  |
| 178.47 | 6.68 | 0.23 |  |  |
| 180.03 | 17.21 | 0.02 |  |  |
| 182.14 | 36.46 | 0.08 |  |  |
| 182.23 | 46.66 | 1.35 |  |  |
| 183.74 | 5.54 | 0.17 |  |  |
| 187.10 | 3.37 | 0.09 |  |  |
| 187.40 | 3.98 | 0.11 |  |  |

**Supplementary Table 3.** The Fe/Al mass ratios for TCDP core 3 originally reported in Cheng et al., 2019^2^.

| **Depth (m)** | Fe/Al mass |
| --- | --- |
| **76.3** | 0.66 |
| **79.15** | 0.67 |
| **82.51** | 0.62 |
| **86.28** | 0.56 |
| **89.1** | 0.7 |
| **91.7** | 0.63 |
| **93.5** | 0.64 |
| **95** | 0.65 |
| **98.44** | 0.59 |
| **100.57** | 0.61 |
| **102.68** | 0.6 |
| **103.35** | 0.59 |
| **105.25** | 0.63 |
| **107.8** | 0.65 |
| **108.65** | 0.6 |
| **109.8** | 0.65 |
| **115.14** | 0.55 |
| **116.37** | 0.59 |
| **118.27** | 0.54 |
| **120.78** | 0.57 |
| **123.88** | 0.56 |
| **126.29** | 0.55 |
| **129.47** | 0.51 |
| **135.22** | 0.52 |
| **141.71** | 0.57 |
| **141.71** | 0.61 |
| **144.4** | 0.62 |
| **144.8** | 0.86 |
| **149.19** | 0.78 |
| **151.57** | 0.7 |
| **151.57** | 0.68 |
| **153.29** | 0.82 |
| **154.97** | 0.73 |
| **155.02** | 0.59 |
| **155.02** | 0.6 |
| **155.13** | 0.59 |
| **156.54** | 0.83 |
| **157.3** | 0.6 |
| **157.3** | 0.74 |
| **161.2** | 0.79 |
| **161.2** | 0.77 |
| **161.39** | 0.8 |
| **162.29** | 0.82 |
| **164.39** | 3.02 |
| **164.54** | 5.1 |
| **168.6** | 0.57 |
| **170.64** | 0.57 |
| **171.68** | 0.62 |
| **171.75** | 0.56 |
| **174.22** | 1.09 |
| **182.22** | 6.04 |
| **182.25** | 21.94 |
| **183.73** | 0.37 |
| **186.98** | 0.5 |

**Supplementary Table 4.** Sulphate δ^18^O isotope standards measured for correction during sample sessions. The target value for NBS-127 was 9.3 ±0.4‰ with respect to VSMOW.

| **Standard** | **δ^18^O (‰ VSMOW)** |
| --- | --- |
| NBS-127 | 9.992 |
| NBS-127 | 9.438 |
| NBS-127 | 9.460 |
| NBS-127 | 9.327 |
| NBS-127 | 9.116 |
| NBS-127 | 9.300 |
| NBS-127 | 9.300 |
| NBS-127 | 9.300 |
| NBS-127 | 9.300 |
| NBS-127 | 9.300 |
| NBS-127 | 9.300 |
| NBS-127 | 9.300 |
| NBS-127 | 9.300 |
| NBS-127 | 9.300 |
| NBS-127 | 9.300 |
| NBS-127 | 9.300 |
| NBS-127 | 9.300 |
| NBS-127 | 9.154 |
| NBS-127 | 9.278 |
| NBS-127 | 9.456 |
| NBS-127 | 9.396 |
| average | 9.344 |
| 1sd | 0.165 |

**Supplementary Table 5.** Multiple sulphur isotope standard measured for correction during sample sessions. The target values for IAEA-S-1 were ∆^33^S = 0.082‰, ∆^36^S = –0.897‰, and δ^34^S = –0.30‰ versus VCDT. The reported uncertainties (2σ) ±0.01‰, ±0.2‰, and ±0.1‰ for ∆^33^S, ∆^36^S, and δ^34^S, respectively, are based on the long-term reproducibility of this standard versus the in-house reference gas.

| **Standard** | **∆^33^S (‰)** | **∆^36^S (‰)** | **δ^34^S (‰ VCDT)** |
| --- | --- | --- | --- |
| IAEA-S-1 | 0.074 | -0.862 | -0.390 |
| IAEA-S-1 | 0.084 | -0.750 | -0.459 |
| IAEA-S-1 | 0.114 | -0.919 | -0.283 |
| IAEA-S-1 | 0.083 | - | -0.340 |
| IAEA-S-1 | 0.087 | -0.747 | -0.398 |

Supplementary Note 1

**Sulphur isotope, ∆^33^S, compilation sources**

Figure 1a, the graph of sulphur ∆^33^S data over time, uses data sourced mainly from two compilations: from Havig et al. (2017)^3^, with data removed from two studies reporting sulphur from non-sedimentary processes of hydrothermal^4^ and thermochemical sulphate reduction^5^, and with ∆^33^S values correctly compiled for one study^6^ whose δ^33^S were accidently substituted for ∆^33^S; and from Williford et al., (2011)^7^ with an adjustment to the dates (from 2.4 Gyr ago to 2.45 Gyr ago) for the original data reported for the Boolgeeda formation in ref. ^7^ in light of new age constraints and correlation by Philippot et al. (2018)^1^; and additional ∆^33^S data sources^1,8-13^.

**Oxygen isotope, δ^18^O, compilation sources**

Figure 1b, the graph of sedimentary δ^18^O data over time, uses a compilation of chert, carbonate, and shale given by Bindeman et al. (2016)^14^, with an additional chert dataset^9^, data for the Kazput carbonate^15^, and sulphate data^13,16-21^

**Supplementary References**

1 Philippot, P. *et al.* Globally asynchronous sulphur isotope signals require re-definition of the Great Oxidation Event. *Nature Communications* **9**, 2245 (2018). <<https://www.nature.com/articles/s41467-018-04621-x>>.

2 Cheng, C. *et al.* Nitrogen isotope evidence for stepwise oxygenation of the ocean during the Great Oxidation Event. *Geochim. Cosmochim. Acta* (2019).

3 Havig, J. R., Hamilton, T. L., Bachan, A. & Kump, L. R. Sulfur and carbon isotopic evidence for metabolic pathway evolution and a four-stepped Earth system progression across the Archean and Paleoproterozoic. *Earth-Science Reviews* (2017).

4 Golding, S. D. *et al.* in *Earliest Life on Earth: Habitats, Environments and Methods of Detection* (eds S. D. Golding & M. Glikson) 15-49 (Springer 2011).

5 Young, S. A., Loukola-Ruskeeniemi, K. & Pratt, L. M. Reactions of hydrothermal solutions with organic matter in Paleoproterozoic black shales at Talvivaara, Finland: Evidence from multiple sulfur isotopes. *Earth Planet. Sci. Lett.* **367**, 1-14 (2013).

6 Paris, G., Adkins, J. F., Sessions, A. L., Webb, S. M. & Fischer, W. W. Neoarchean carbonate-associated sulfate records positive ∆33S anomalies. *Science* **346**, 739-741 (2014).

7 Williford, K. H., Van Kranendonk, M. J., Ushikubo, T., Kozdon, R. & Valley, J. W. Constraining atmospheric oxygen and seawater sulfate concentrations during Paleoproterozoic glaciation: In situ sulfur three-isotope microanalysis of pyrite from the Turee Creek Group, Western Australia. *Geochim. Cosmochim. Acta* **75**, 5686-5705 (2011).

8 Luo, G. *et al.* Rapid oxygenation of Earth’s atmosphere 2.33 billion years ago. *Science Advances* **2**, e1600134 (2016). <<https://advances.sciencemag.org/content/2/5/e1600134>>.

9 Kitayama, Y., Thomassot, E., O'Neil, J. & Wing, B. A. Sulfur- and oxygen-isotope constraints on the sedimentary history of apparent conglomerates from the Nuvvuagittuq Greenstone Belt (Nunavik, Québec). *Earth Planet. Sci. Lett.* **355-356**, 271-282 (2012).

10 Izon, G. *et al.* Multiple oscillations in Neoarchaean atmospheric chemistry. *Earth Planet. Sci. Lett.* (2015).

11 Izon, G. *et al.* Biological regulation of atmospheric chemistry en route to planetary oxygenation. *Proceedings of the National Academy of Sciences* **114**, E2571-E2579 (2017).

12 Muller, É. *et al.* Primary sulfur isotope signatures preserved in high-grade Archean barite deposits of the Sargur Group, Dharwar Craton, India. *Precambrian Res.* **295**, 38-47 (2017).

13 Crockford, P. W. *et al.* Claypool continued: Extending the isotopic record of sedimentary sulfate. *Chem. Geol.* (2019).

14 Bindeman, I. N., Bekker, A. & Zakharov, D. O. Oxygen isotope perspective on crustal evolution on early Earth: A record of Precambrian shales with emphasis on Paleoproterozoic glaciations and Great Oxygenation Event. *Earth Planet. Sci. Lett.* **437**, 101-113 (2016).

15 Barlow, E., Van Kranendonk, M., Yamaguchi, K., Ikehara, M. & Lepland, A. Lithostratigraphic analysis of a new stromatolite–thrombolite reef from across the rise of atmospheric oxygen in the Paleoproterozoic Turee Creek Group, Western Australia. *Geobiology* **14**, 317-343 (2016).

16 Claypool, G. E., Holser, W. T., Kaplan, I. R., Sakai, H. & Zak, I. The age curves of sulfur and oxygen isotopes in marine sulfate and their mutual interpretation. *Chem. Geol.* **28**, 199-260 (1980).

17 Bao, H., Rumble, D. & Lowe, D. R. The five stable isotope compositions of Fig Tree barites: Implications on sulfur cycle in ca. 3.2 Ga oceans. *Geochim. Cosmochim. Acta* **71**, 4868-4879 (2007).

18 Bao, H., Lyons, J. R. & Zhou, C. Triple oxygen isotope evidence for elevated CO2 levels after a Neoproterozoic glaciation. *Nature* **453**, 504-506, doi:10.1038/nature06959 (2008).

19 Peng, Y., Bao, H., Zhou, C., Yuan, X. & Luo, T. Oxygen isotope composition of meltwater from a Neoproterozoic glaciation in South China. *Geology* **41**, 367-370 (2013).

20 Turchyn, A. V. & Schrag, D. P. Oxygen isotope constraints on the sulfur cycle over the past 10 million years. *Science* **303** (2004).

21 Crockford, P. W. *et al.* Triple oxygen isotope evidence for limited mid-Proterozoic primary productivity. *Nature* (2018).
