# Supplementary material for: Constraining the rise of oxygen with oxygen isotopes
Source: Nat Commun. 2019 Oct 29;10:4924. doi: 10.1038/s41467-019-12883-2 (PMC6820740; doi:10.1038/s41467-019-12883-2)
Supplement: Supplementary file 3 — Reporting Summary [file 41467_2019_12883_MOESM3_ESM.pdf]

nature research

Corresponding author(s): Bryan Killingsworth

Last updated by author(s): Oct 2, 2019

# Reporting Summary

Nature Research wishes to improve the reproducibility of the work that we publish. This form provides structure for consistency and transparency in reporting. For further information on Nature Research policies, see Authors & Referees and the Editorial Policy Checklist.

## Statistics

For all statistical analyses, confirm that the following items are present in the figure legend, table legend, main text, or Methods section.

| n/a | Confirmed | |
|---|---|---|
| ☐ | ☒ | The exact sample size (*n*) for each experimental group/condition, given as a discrete number and unit of measurement |
| ☐ | ☒ | A statement on whether measurements were taken from distinct samples or whether the same sample was measured repeatedly |
| ☒ | ☐ | The statistical test(s) used AND whether they are one- or two-sided<br>*Only common tests should be described solely by name; describe more complex techniques in the Methods section.* |
| ☒ | ☐ | A description of all covariates tested |
| ☒ | ☐ | A description of any assumptions or corrections, such as tests of normality and adjustment for multiple comparisons |
| ☒ | ☐ | A full description of the statistical parameters including central tendency (e.g. means) or other basic estimates (e.g. regression coefficient) AND variation (e.g. standard deviation) or associated estimates of uncertainty (e.g. confidence intervals) |
| ☒ | ☐ | For null hypothesis testing, the test statistic (e.g. *F*, *t*, *r*) with confidence intervals, effect sizes, degrees of freedom and *P* value noted<br>*Give P values as exact values whenever suitable.* |
| ☒ | ☐ | For Bayesian analysis, information on the choice of priors and Markov chain Monte Carlo settings |
| ☒ | ☐ | For hierarchical and complex designs, identification of the appropriate level for tests and full reporting of outcomes |
| ☒ | ☐ | Estimates of effect sizes (e.g. Cohen's *d*, Pearson's *r*), indicating how they were calculated |

*Our web collection on statistics for biologists contains articles on many of the points above.*

## Software and code

Policy information about availability of computer code

| | |
|---|---|
| Data collection | Isodat, MS IonVantage |
| Data analysis | Microsoft Excel, R |

For manuscripts utilizing custom algorithms or software that are central to the research but not yet described in published literature, software must be made available to editors/reviewers. We strongly encourage code deposition in a community repository (e.g. GitHub). See the Nature Research guidelines for submitting code & software for further information.

## Data

Policy information about availability of data

All manuscripts must include a data availability statement. This statement should provide the following information, where applicable:

- Accession codes, unique identifiers, or web links for publicly available datasets
- A list of figures that have associated raw data
- A description of any restrictions on data availability

All data generated or analyzed during this study are included in this published article (and its supplementary information files).

# Field-specific reporting

Please select the one below that is the best fit for your research. If you are not sure, read the appropriate sections before making your selection.

☐ Life sciences ☐ Behavioural & social sciences ☒ Ecological, evolutionary & environmental sciences

For a reference copy of the document with all sections, see nature.com/documents/nr-reporting-summary-flat.pdf

# Ecological, evolutionary & environmental sciences study design

All studies must disclose on these points even when the disclosure is negative.

| | |
|---|---|
| Study description | Sulfur and oxygen stable isotope study of sulfate, specifically barite, found in rocks of <2.31 billion years age. Isotope parameters measured include Δ33S, Δ36S, d34S for sulfur, and d18O for oxygen. |
| Research sample | Sedimentary rock samples from drill core from Western Australia that were previously collected as part of the Turee Creek Drilling Project headed by Pascal Philippot (co-author of this present study). |
| Sampling strategy | Sufficient samples were chosen to represent any stratigraphic variation on the bulk sample scale, at roughly 1 sample per 1 meter depth in the drill core. Approximately 200g over 10 cm was taken for each sample. |
| Data collection | Sulfur isotope data was measured by Bryan Killingsworth at IPGP, France. Oxygen isotope data was measured by Bryan Killingsworth with the assistance of the engineer Théophile Cocquerez at the University of Bourgogne, Dijon, France. |
| Timing and spatial scale | There is no time-dependent sampling in these rock samples. The spatial scale is one 17 cm long (on average) sample of approximately 200g weight taken per meter of sedimentary rock drill core. |
| Data exclusions | No S or O isotope data were excluded from the reported data, therefore the sampling was continuous with respect to the spatial/depth scale used. |
| Reproducibility | All S isotope measurements were done singly, where S isotope standards were measured in the same sessions to ensure accuracy. All O isotope measurements were done in duplicate. |
| Randomization | Randomization is not relevant to our study, as it is an Earth Sciences study on samples whose reporting by stratigraphic sequence is relevant to their understanding in time relative to each other. |
| Blinding | Blinding was not relevant to this Earth Sciences study. |

Did the study involve field work? ☐ Yes ☒ No

# Reporting for specific materials, systems and methods

We require information from authors about some types of materials, experimental systems and methods used in many studies. Here, indicate whether each material, system or method listed is relevant to your study. If you are not sure if a list item applies to your research, read the appropriate section before selecting a response.

## Materials & experimental systems

| n/a | Involved in the study | |
|---|---|---|
| ☒ | ☐ | Antibodies |
| ☒ | ☐ | Eukaryotic cell lines |
| ☒ | ☐ | Palaeontology |
| ☒ | ☐ | Animals and other organisms |
| ☒ | ☐ | Human research participants |
| ☒ | ☐ | Clinical data |

## Methods

| n/a | Involved in the study | |
|---|---|---|
| ☒ | ☐ | ChIP-seq |
| ☒ | ☐ | Flow cytometry |
| ☒ | ☐ | MRI-based neuroimaging |
